# Effect of Cutaneous Heat Pain on Corticospinal Excitability of the Tibialis Anterior at Rest and during Submaximal Contraction

**DOI:** 10.1155/2018/8713218

**Published:** 2018-04-26

**Authors:** Maxime Billot, Cécilia Neige, Martin Gagné, Catherine Mercier, Laurent J. Bouyer

**Affiliations:** ^1^Department of Rehabilitation, Université Laval, 1050 Avenue de la Médecine, Quebec City, QC, Canada G1V 0A6; ^2^Center for Interdisciplinary Research in Rehabilitation and Social Integration (CIRRIS), 525 Boul. Wilfrid-Hamel, Quebec City, QC, Canada G1M 2S8

## Abstract

Previous studies have shown that pain can interfere with motor control. The neural mechanisms underlying these effects remain largely unknown. At the upper limb, mounting evidence suggests that pain-induced reduction in corticospinal excitability is involved. No equivalent data is currently available at the lower limb. The present study therefore examined the effect of thermal pain on the corticospinal drive to tibialis anterior (TA) at rest and during an isometric submaximal dorsiflexion. Transcranial magnetic stimulation was used to induce motor-evoked potentials (MEPs) in the TA at rest and during contraction in the presence or absence of cutaneous heat pain induced by a thermode positioned above the TA (51°C during 1 s). With similar pain ratings between conditions (3.9/10 at rest and 3.6/10 during contraction), results indicate significant decreases in MEP amplitude during both rest (−9%) and active conditions (−13%) (main effect of pain, *p* = 0.02). These results therefore suggest that cutaneous heat pain can reduce corticospinal excitability in the TA muscle and that such reduction in corticospinal excitability could contribute to the interference of pain on motor control/motor learning.

## 1. Introduction

Alterations in sensory inputs, such as cutaneous stimulation, deafferentation, or pain, can affect the motor system and motor control in various ways [1–3]. Converging lines of evidence show that pain can interfere with motor performance [4–7], but findings from motor learning studies are mixed, showing that motor learning can be either disrupted [8, 9] or conversely improved [2, 10, 11] in the presence of pain. In fact, the interactions between pain and motor learning appear to vary according to the type of task (e.g., gait [12, 13], reaching [14], and manual tasks [9–11, 15–17]), or according to the muscles involved [7]. This suggests that the impact of pain on the motor system might vary according to the limb/muscle and to the type of activity.

Transcranial magnetic stimulation (TMS) has been commonly used to investigate the effect of experimental pain on corticospinal excitability. Together, TMS studies show no consensus and it has been reported that pain can either induce a decrease [18–24] and increase [25–27] or have no effect [16, 28–30] on corticospinal excitability. This might be attributed, at least in part, to several methodological differences between studies. First, different limbs have been tested to investigate the effect of pain on corticospinal excitability. To date, several TMS studies have reported data from the upper limb [18, 21, 25, 28–30], whereas only one reported results for the lower limb [26]. Importantly, while most studies targeting the upper limb reported a decrease in corticospinal excitability, the study focusing on the lower limb reported an increase in corticospinal excitability. It is also important to note that for the upper limb, pain induced an increase in corticospinal excitability for proximal muscles such as biceps brachii and a decrease in corticospinal excitability for distal hand muscles [31].

Another potential source of variability that might contribute to the heterogeneity between studies is the difference in pain intensity; unfortunately, individual pain ratings were seldom reported [22, 23, 32, 33]. One study reported a lack of correlation between pain intensity and the modulation of corticospinal excitability [18], but this is insufficient to generalize given the small sample size and the range of pain intensities used.

Finally, the state of the target muscle (rest versus contraction) is an important factor to consider when investigating the modulation of corticospinal excitability [34]. Most studies on the effect of pain on corticospinal excitability have been performed with the target muscle at rest [18, 19, 26, 28, 35–37]. A few other studies have been performed with the target muscle slightly contracted [25, 29, 30, 38], and only two studies tested target muscles both at rest and during contraction [27, 39]. In their study, Martin et al. [39] reported that for triceps brachii, corticospinal excitability was not modulated by pain at rest but was reduced during contraction. In a recent meta-analysis, Burns et al. [40] reported no significant effect of experimental pain on corticospinal excitability during active muscle contraction, contrary to the moderate effect induced by pain in the same muscle measured at rest. The authors suggest that the absence of effect could be due to the facilitatory influence of volitional contraction on MEP amplitude making modulation by pain undetectable [40].

To address some of the gaps identified in our understanding of the effect of pain on the motor system, the main goal of this study was to determine the effect of pain on corticospinal excitability of a lower-limb muscle, the tibialis anterior (TA), tested both at rest and under active contraction. As TA is a distal muscle, it was hypothesized that pain would decrease corticospinal excitability, as reported for distal upper-limb muscles. Moreover, it was hypothesized that the effect of pain would be larger at rest than during contraction, due to a masking effect of volitional contraction on MEP amplitude. As a secondary objective, the relationship between pain intensity and MEP amplitude modulation was also explored.

## 2. Methods

### 2.1. Participants

Eighteen young adults (10 women; age: 24.6 ± 5.4 years) volunteered for this two-session study. They had no reported history of neurological or orthopaedic problems affecting the lower limb, nor chronic/acute pain prior to the experiment. All participants provided written informed consent to the study. This experiment was approved by the institutional ethics review board (CER #2010-212) and is in accordance with the Declaration of Helsinki.

### 2.2. General Protocol

**Figure 1:**
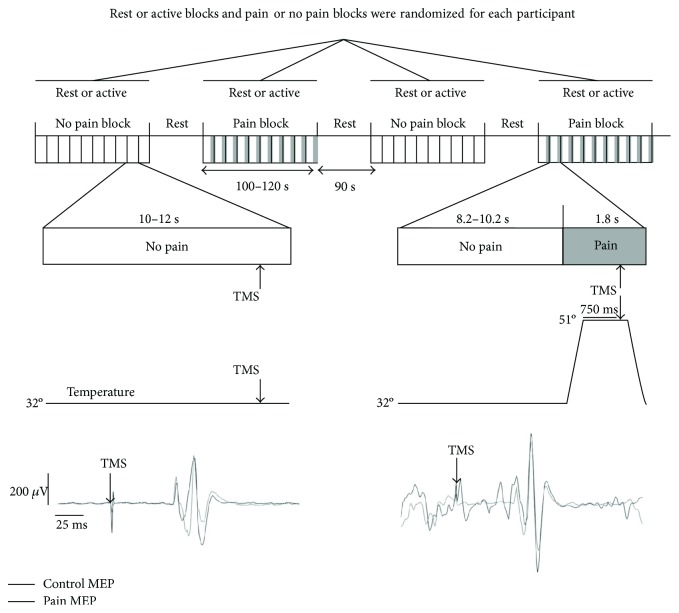
Experimental protocol in pain or no pain blocks at rest or during dorsiflexion (10% of MVC). Ten stimulations were evoked by block, and the blocks were randomized. A rest period of 90 s was given between blocks. TMS was triggered 750 ms after the plateau temperature was reached.

Figure 1 illustrates the experimental protocol. Participants took part in two experimental sessions, separated by one week, one with the muscle tested at rest (rest condition) and the other with the muscle tested during submaximal contraction (active condition). Order of the sessions was counterbalanced across participants. In each session, TMS and thermal nociceptive stimulation intensities were determined (see below), and four randomized blocks of 10 MEPs were recorded: two blocks with (pain condition) and two blocks without (control condition) thermal nociceptive stimulation, for a total of 20 MEPs per condition. During the active condition session, electromyographic (EMG) activity during TA maximal voluntary contraction (MVC) was quantified prior to MEP data collection. The dominant limb was assessed in all participants by stimulating the left motor cortex for right-hand dominants (*n* = 16) and the right motor cortex for left-hand dominants (*n* = 2).

### 2.3. Recordings

Participants were seated in a Biodex System 3 ergometer (Shirley, NY Biodex 3) with the hip joint at 80°, the knee joint flexed at 60°, and the ankle in neutral position. The right foot was fixed to a footplate using straps. To minimize trunk and hip movements during contraction, the waist was stabilized by means of a belt and arms were positioned across the chest. Participants were instructed to fix their gaze on a target (rest condition) or on a computer screen (active condition) located at the eye level, 1.5 m away.

The TA was selected as the target muscle due to its important corticospinal drive [41], thereby being a good model to assess pain-related modulation of corticospinal excitability at the lower limb. EMG activity was recorded using Ag/AgCl disposable surface electrodes (Kendall Medi-Trace 200, Covidien). Electrode placement followed SENIAM recommendations (Hermens et al. 2000). A ground electrode was placed on the patella. Low impedance at the skin-electrode interface was obtained by shaving and cleaning the skin with alcohol. EMG signals were amplified and band-pass filtered (20–1000 Hz) and sampled at 2000 Hz (CED 1401 interface; Cambridge Electronic Design, Cambridge, UK).

### 2.4. MVC and Submaximal Contraction

TA EMG activity was recorded during two 5-6 s isometric MVCs in dorsiflexion. TA EMG_max_ was defined as the maximal root mean square (RMS) EMG value of the two MVCs. The active condition consisted in maintaining an isometric dorsiflexion at 10% (±2%) of TA EMG_max_. During the active condition, EMG feedback was provided on a computer screen (500 ms moving average window). The level of submaximal contraction was similar to that measured during gait [42].

### 2.5. TMS

First, the TA hotspot (i.e., scalp site where responses were evoked at the lowest intensity of stimulation) over the primary motor cortex was defined and registered using a neuronavigation system (Brainsight, Rogue Research Inc., Montreal, Canada). To ensure consistent stimulation location, the neuronavigation system was used throughout the experiment. A custom bat-wing-shaped figure-of-eight coil (wing diameter of 90 mm; The Magstim Co., Whitland, Dyfed, UK) placed in anterior-posterior direction was used to elicit motor-evoked potentials (MEPs) in the TA muscle. The motor threshold (MT) was measured at rest (rMT) and at 10% of TA EMG_max_ (aMT), depending on the experimental session. MT was defined as the minimal intensity of stimulation required to elicit MEPs larger than 50 *μ*V (rest condition) or clearly discernable above baseline EMG background (active condition) in three out of six stimulations. An intensity of 120% MT (of rMT for the rest condition and of aMT for the active condition) was then used during the experiment. When MEPs were less than 300 *μ*V at 120% of MT (occurring in five participants in the rest condition and four participants in the active condition), stimulation intensity was increased (mean: 123.6% (range 120–135%) in the rest condition; 122.4% (range 120–136%) in the active condition). In three participants, no MEPs were obtained at rest, even at 100% of maximal stimulator output, and therefore had to be rejected. Consequently, only data from 15 participants were included in the analysis (9 women; age: 24.7 ± 5.4 years).

### 2.6. Nociceptive Stimulus

A thermode (CHEPS-2001, Medoc Ltd.; 27 mm diameter) was used to evoke pain. Participants were informed that the stimuli would not cause skin damage. Contact between the thermode and the skin was maintained using an elastic band positioned around the leg. The thermal nociceptive stimulus consisted of a 400 ms ramp-up to 51°C, followed by a 1 s plateau and a 400 ms ramp-down. After each experimental block, subjects were asked to rate pain intensity on a numerical rating scale ranging from 0 (no pain) to 10 (maximal pain that they could imagine).

To ensure that nociceptive afferent information from the lower limb had reached the cortex prior to evoking MEPs (SEP latency to thermal pain stimulation = 532 ± 19 ms [43]), TMS was triggered with a 750 ms delay after the onset of the thermode plateau temperature. To minimize anticipation, the interval between TMS pulses was jittered (see Figure 1). Experimental blocks were separated by 90 s rest periods. The temperature had to be reduced in one participant in which 51°C was not tolerable (reduced to 49.5°C, leading to a pain rating of 7/10).

### 2.7. Data Analysis

Mean of the peak-to-peak amplitude of the 20 MEPs per condition (control versus pain; rest versus active) was used for analysis, for a total of 80 MEPs per participant. A percentage of pain-induced corticospinal excitability modulation was calculated for each participant in each muscle condition as (mean MEP in pain condition − mean MEP in control condition)∗100/mean MEP in control condition. At rest, trials where EMG activity was visually detected were excluded from the analysis. In addition, background EMG was quantified for each trial using a 100 ms window preceding each MEP (mean RMS value). For the active condition, only trials where the background EMG activity was within 2% of the 10% of MVC target were kept for analysis. The trials where background EMG was greater than three standard deviations from the mean were also rejected. A total of 75.5% of all MEPs were kept for analysis, with no difference across conditions.

### 2.8. Statistics

All data are presented as means ± standard deviations for the 15 participants. A two-way repeated measure ANOVA analysis [condition (rest or active) × pain (control or pain), 2 × 2] was performed on the MEPs. The relationship between MEP modulation and pain intensity, for both rest and active conditions, was assessed using Pearson's coefficient of correlation. Finally, additional analyses were performed to control for potential methodological biases. To do so, the RMS values and the numerical scale scores were compared using a two-way repeated measure ANOVA [condition (rest or active) × pain (control or pain)] or a paired *t*-test (rest versus active, pain condition only), respectively. All statistical analyses were performed using version 22 of SPSS software (SPSS Inc., Chicago, IL, USA). Statistical significance was set at *p* < 0.05.

## 3. Results

### 3.1. Pain Ratings and Background EMG Levels across Conditions

**Figure 2:**
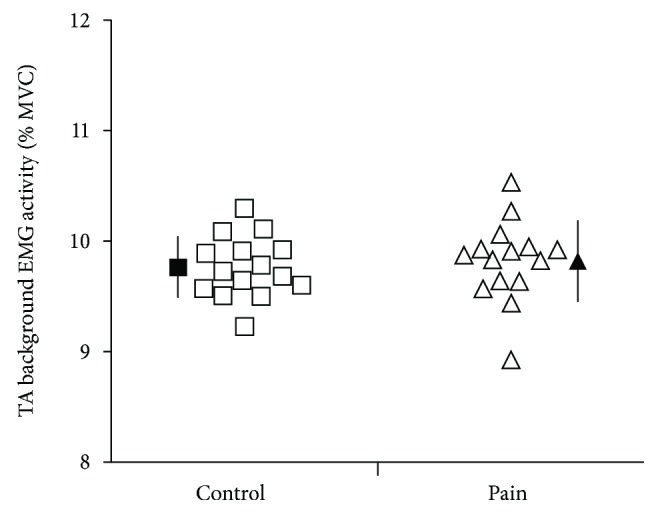
TA background EMG activity (%MVC) 200 ms before TMS, with and without pain for each participant (open symbol) and means of all participants (black symbols). With a target of 10% MVC, all trials kept for analysis were between 8% and 12%.

The thermal nociceptive stimuli induced an average pain rating of 3.9 ± 1.8 for the rest condition and 3.6 ± 1.8 for the active condition. There was no significant difference across conditions (*p* = 0.50). Unsurprisingly, analysis revealed that the mean TA background EMG activity (RMS value) was significantly higher during the active condition compared to the rest condition (*p* < 0.001). As illustrated in Figure 2, there was no significant difference between the pain and control conditions (*p* = 0.13) or condition × pain interaction (*p* = 0.45) directly, however, indicating that pain did not affect background EMG (which may otherwise have biased MEP results).

### 3.2. Effect of Pain on Corticospinal Excitability with the Target Muscle at Rest versus Active

**Figure 3:**
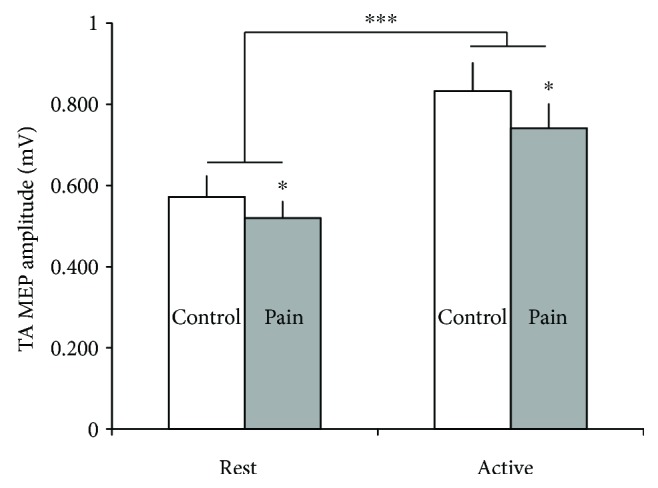
Mean TA MEP amplitude at rest and during active conditions, without pain (control in white) and with pain (in grey). Error bars represent the standard error of the mean (SEM). ^∗^*p* < 0.05; ^∗∗∗^*p* < 0.001.

Figure 3 shows the TA MEP mean amplitudes with the target muscle at rest versus active, in both control and pain conditions. There was a significant main effect of muscle condition (*F*(1,14) = 17.6, *p* = 0.001) and of pain (*F*(1,14) = 5.3, *p* = 0.037) on TA MEPs, without any significant interaction (*F*(1,14) = 0.4, *p* = 0.853). Unsurprisingly, TA MEPs were larger during the active (0.80 ± 0.24 mV) than during the rest condition (0.55 ± 0.18 mV; *F*(1,14) = 17.6, *p* = 0.001). Furthermore, results indicate that TA MEPs were smaller in the pain (0.52 ± 0.16 mV at rest and 0.76 ± 0.23 mV in active) than in the control condition (0.57 ± 0.20 mV at rest and 0.83 ± 0.27 mV in active; *F*(1,14) = 5.3, *p* = 0.037).

**Figure 4:**
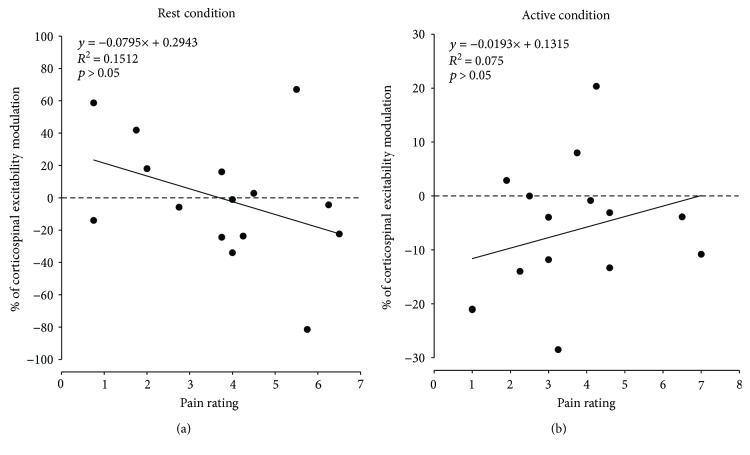
Association between percentage of change in TA MEPs and pain rating for both rest (a) and active conditions (b). Each dot represents a participant. Data above the dashed line represents corticospinal facilitation, and data below the dashed line represents corticospinal inhibition. There was no correlation between corticospinal modulation and pain ratings.

Finally, as illustrated in Figure 4, no significant correlation was observed between the pain-induced modulation of MEPs and the intensity of pain, either in the rest (Figure 4(a)) or in the active condition (Figure 4(b); all *p* > 0.05).

## 4. Discussion

The main objective of this study was to investigate the effect of pain on corticospinal excitability in the TA muscle, at rest and during submaximal contraction.

The first result of this study is, as hypothesized, that thermal pain induced a decrease in corticospinal excitability. This finding is consistent with previous studies in hand muscles [18–24, 28]. In 1999, Valeriani et al. [22] proposed the “motor decerebration” hypothesis to illustrate the observed inhibition of corticospinal excitability induced by pain. This inhibition may be related to protective withdrawal reflexes acting at both spinal and supraspinal sites to protect the organism [18, 30, 32, 40]. However, the findings of the current study are opposite to those of the only other study that investigated the lower limb [26]. By recording MEPs in quadriceps after injection of hypertonic saline into the infrapatellar fat pad at rest, Rice et al. [26] found that pain significantly increased MEP amplitude in the vastus lateralis, whereas no changes were observed in the biceps femoris or TA. Two factors could account for this difference with our results. First, Rice et al. [26] used a hypertonic saline injection to induce tonic experimental pain, whereas we used thermal skin stimulations to induce phasic experimental pain. As suggested by Farina et al. [20], interactions between the nociceptive and motor systems might differ according to the duration (tonic versus phasic) and the modality (deep versus superficial) of pain, due to their different spinal and supraspinal processing. Secondly, these authors investigated a proximal muscle, while a distal muscle was used in the present study. According to Kofler et al. [31], proximal and distal muscles might be differentially modulated by nociceptive stimuli. They suggest that pain would induce a complex protective reflex where corticospinal excitability of distal painful muscles becomes inhibited and proximal muscles involved in withdrawal become facilitated. Accordingly, electroencephalography (EEG) studies demonstrated that the application of heat nociceptive stimuli results in decreased M1 *β* activity [44, 45]. The inhibitory role of *β* activity in the motor cortex [46] suggests that pain reduces inhibition to facilitate withdrawal responses of proximal muscles. In the current study, pain was induced over the TA muscle, not over the foot, making a direct comparison impossible. Further studies will be required to assess the effect of pain on proximal versus distal muscles at the lower limb according to pain location.

In the current study, no correlation was found between the pain intensity reported by participants and changes in TA corticospinal excitability. This result suggests that corticospinal excitability was independent of subjective pain perception, in accordance with a previous study using a very similar method for hand muscles [18]. In this previous study, the mean intensity of the perceived pain (2.8/10 ± 1.9 on the numerical rating scale) was smaller compared to that in the current study. The average pain-induced MEP amplitude reduction was between 18 and 25% at the hand at rest and between 9 and 13% in TA in the present study. This finding suggests that the modulation of corticospinal excitability induced by pain for the hand might be superior to that for the leg, despite the fact that pain was higher at the leg. A possible explanation for this result could be the greater number of sensory fibers and larger somatosensory cortical representation for the hand than for the leg. However, an alternative explanation could be that large modulations of corticospinal excitability are more difficult to induce in lower-limb muscles as their input-output curves are not as steep as those for hand muscles [47, 48]. The current study being only correlational, further studies should use different levels of nociceptive inputs to address whether pain intensity affects MEP modulation.

The second aim of this study was to compare the effect of pain on TA corticospinal excitability at rest to that during contraction. In line with a recent systematic review [40] in which authors suggested that muscle contraction would mask the modulatory effect of pain on the corticospinal system, we initially hypothesized that the effect of pain would be larger at rest than during contraction. However, contrary to our hypothesis, results showed that the effect of pain did not differ between these conditions. Despite no significant difference in the pain-induced modulation of MEPs between active and rest condition, it seems that the decrease in corticospinal excitability was observed more systematically among the participants during the active condition than during the rest condition (see Figure 4(a) and 4(a)). One possible explanation for this result is that contracting the target muscle during the corticospinal excitability investigation leads to a stabilization of the motoneuronal pool and also allowed evoking MEPs more easily. Further studies are needed to address this underlying mechanism.

## 5. Conclusion

The current study showed that heat pain applied at the lower limb significantly reduced TA corticospinal excitability during rest and submaximal dorsiflexion. If the functional impact of pain on motor control and motor learning is still debated in the literature, the results of the present study provide further evidence that nociceptive sensory input can impact corticospinal excitability.
